# Successful radiofrequency thermocoagulation of the mandibular nerve for intractable pain associated with medication-related osteonecrosis of the jaw: a case report

**DOI:** 10.1186/s40981-024-00696-2

**Published:** 2024-02-13

**Authors:** Sho Shinotsuka, Aiko Maeda, Tomoka Eri, Nozomi Kameyama, Chiaki Yamada, Masako Asada, Ken Yamaura

**Affiliations:** 1 Department of Anesthesiology and Critical Care Medicine, Kyushu University Hospital, 3-1-1 Maidashi Higashi-Ku, Fukuoka City, Fukuoka, 812-8582 Japan; 2 Operating Rooms, Kyushu University Hospital, Fukuoka, Japan; 3 Department of Anesthesiology and Critical Care Medicine, Kyushu University Graduate School of Medicine, Fukuoka, Japan

**Keywords:** Mandibular nerve block, Radiofrequency thermocoagulation, Medication-related osteonecrosis of the jaw

## Abstract

**Background:**

Bisphosphonates may cause serious adverse events, including osteonecrosis of the jaw. This article describes a case of successful application of radiofrequency thermocoagulation for pain caused by osteonecrosis of the jaw.

**Case presentation:**

An 86-year-old woman who had received alendronate sodium hydrate for osteoporosis was diagnosed with osteonecrosis of the right mandible after dental treatment. Despite repeated conservative and debridement treatments, the patient could not eat due to intractable pain; accordingly, her condition was debilitated. The patient was referred to our pain management clinic for radiofrequency thermocoagulation of the right mandibular nerve. Immediately after the procedure, her pain drastically improved and she could eat; moreover, the pain has not recurred for 3 years.

**Conclusion:**

Our findings demonstrate that minimally invasive radiofrequency thermocoagulation may have long-term effects in patients with chronic pain caused by osteonecrosis of the jaw that is refractory to conservative treatment.

## Background

Bisphosphonates and denosumab are widely used to prevent skeletal-related events in patients with osteoporosis or bone cancer metastases. However, their administration is associated with medication-related osteonecrosis of the jaw, with an incidence of 1–15% and 0.001–0.01% in patients with cancer and osteoporosis, respectively [1]. Other risk factors for medication-related osteonecrosis of the jaw include glucocorticoid use, maxillary or mandibular bone surgery, poor oral hygiene, chronic inflammation, diabetes mellitus, and ill-fitting dentures [1]. The aim of managing osteonecrosis of the jaw is to improve various symptoms, including pain, as well as quality of life by preventing the progression of osteonecrosis and controlling infection [2]. Although management of the associated pain is a major treatment goal, its treatment strategies remain unclear.

This article describes a case in which osteonecrosis of the jaw was successfully treated with radiofrequency thermocoagulation (RFT) of the mandibular nerve, and pain refractory to conservative treatment was controlled.

## Case presentation

An 86-year-old woman (height, 130 cm; weight, 36 kg) with a history of osteoporosis had been receiving alendronate, a bisphosphonate, since the age of 77 years. She discontinued alendronate sodium hydrate and underwent extraction of the right lower molar due to periodontitis. However, the swelling and pain in the right mandibular gum persisted. She was referred to the dental surgery department and diagnosed with medication-related osteonecrosis of the jaw based on the observed swelling, pus drainage from the gingival fistula, and osteolysis of the right mandible. The pain persisted even with incisional drainage and debridement of the abscess, multiple sequestrectomy attempts, repeated administration of antibacterial agents, and extraction of the right lower residual molars. Moreover, she showed no response to 25 mg of pregabalin daily, tramadol was gradually increased to 75 mg daily, and acetaminophen was gradually increased to 2650 mg daily, which caused severe side effects such as extreme staggering. Five months after the diagnosis, she was referred to our pain management clinic for pain evaluation and treatment.

She presented with persistent right mandibular pain with a numerical rating scale (NRS; 0–10) score of 3. Additionally, she experienced paroxysmal electrical pain with an NRS score of 5 upon mandibular movements, which lasted several tens of minutes and occurred approximately 20 times per day. Otorhinolaryngological and other cranial nerve examinations did not reveal any abnormal findings. Given her normal white blood cell and neutrophil counts, low C-reactive protein levels, and physical examination findings, systemic inflammation was ruled out. The aforementioned medication therapy only yielded transient beneficial efficacy. Moreover, the dose could not be increased due to side effects. She was unable to chew solid food or sleep well, which led to a gradual weight loss of 6.6 kg, and the pain significantly diminished her quality of life. Therefore, we decided to perform minimally invasive RFT of the right mandibular nerve.

**Fig. 1:**
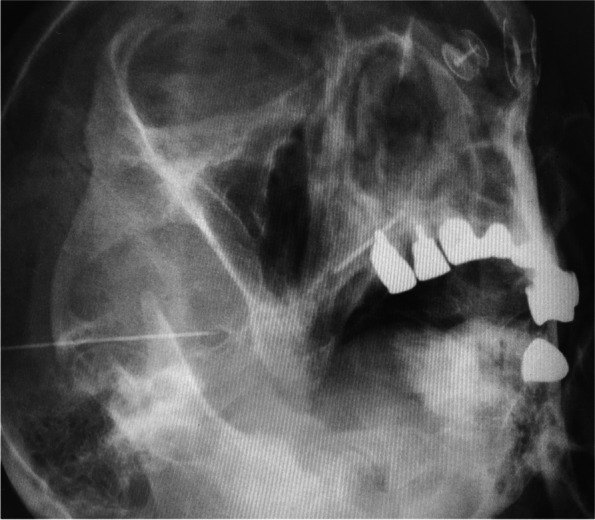
Fluoroscopic image during radiofrequency thermocoagulation of the right mandibular nerve. The tip of the needle coincides with the right foramen ovale

The patient was placed in a supine position with the head slightly bent backward using a shoulder pillow; subsequently, the right foramen ovale was visualized using radiographic fluoroscopy. The puncture point was placed at the midpoint and inferior border of the right zygomatic arch, ≈3 cm ventral to the tragus. After applying local anesthesia with 2 ml of 1% mepivacaine subcutaneously at the puncture site, a 22-gauge, 54-mm radiofrequency needle (TL-S; Top Corporation, Tokyo, Japan) was used to reach the foramen ovale; subsequently, 0.3 V sensory stimulation at 50 Hz was used to produce pain (Fig. 1). Within minutes after injecting 0.5 mL of 2% mepivacaine, sensory loss in the right mandible was obtained. Next, RFT was performed for 180 s at 50 °C (TOP Lesion Generator TLG-10; Top Corporation). The post-interventional course was uneventful without complications. The intractable pain in the right mandible improved immediately after RFT. She experienced a slight numbness in the right mandibular nerve area without masticatory atonia.

She obtained complete pain relief, and all analgesics were discontinued 4 days after the procedure. After receiving appropriate dental treatment for osteonecrosis of the jaw, the patient was discharged from our hospital following a satisfactory treatment course. Her pain was well controlled, with an NRS score of 0 without medication for 3 years. Additionally, she was able to eat meals that she could not before and her weight increased to the original.

This case report complied with the applicable “Improving Quality and Transparency in Health Research” Guidelines. Written informed consent was obtained from the patient for the presentation of this case report.

## Discussion

Ruggiero et al. developed the current clinical staging and treatment strategies for medication-related osteonecrosis of the jaw [3]. Given the presence of pain and erythema in the right mandibular area and exposed necrotic bone associated with infection at the initial stage, our patient was classified as stage 2. Based on the treatment strategies, our patient was treated with systematic antibiotics, incisional drainage, debridement of the abscess, surgical management including removal of the exposed and necrotic bone, and analgesic administration. However, she had persistent severe pain that prevented her from eating and resulted in weight loss, prompting the need for immediate pain relief.

Recent evidence suggests that surgery, including the removal of necrotic bones, can effectively reduce, and ultimately resolve, the pain associated with osteonecrosis of the jaw [2]. However, if the pain has a neurogenic etiology, symptoms may persist Conservative medications are commonly used; non-steroidal anti-inflammatory drugs and acetaminophen for nociceptive pain due to necrosis or infection, pregabalin, mirogabalin, selective serotonin reuptake inhibitors, and weak-to-strong opioids for pain due to neuralgia components [4]. Our patient was refractory to conservative medications and experienced serious adverse effects.

There have been few reports on nonsurgical interventions, other than medication, for pain associated with medication-related osteonecrosis of the jaw. However, neurolysis to the target nerve is occasionally considered effective for chronic neuropathic pain treatment. A previous case report showed that RFT of the Gasserian ganglion improved the quality of life of a patient with medication-related osteonecrosis of the jaw who had terminal cancer [5]. In another report, continuous local anesthetic administration via a catheter near the mandibular nerve has been applied in a patient with medication-related osteonecrosis of the jaw [6]. As for trigeminal neuralgia, RFT is an effective, safe, and minimally invasive neurolysis treatment [7]. Lin et al. performed mandibular nerve block using RFT at 90 °C for 120 s in 104 patients with trigeminal neuralgia and reported a 2-year pain recurrence rate of 8.41% [8]. In our patient, there was no pain recurrence for 3 years, with no complications other than hypoesthesia immediately after the procedure. The improvement in nutritional status due to the ability to eat food and reliable debridement treatment may have contributed to further improvement in her pain. Although RFT causes numbness, which should be explained to the patients for informed consent, other complications are considered less common. Although there are other alternative methods, including surgical neurotomy, minimally invasive RFT should be attempted prior to these methods.

There are currently no clinical studies investigating the efficacy of RFT and the optimal temperature for managing the pain associated with osteonecrosis of the jaw. However, Tun et al. performed ultrastructural evaluation in rat sciatic nerves treated with RF at 42 °C and reported that nearly one-third of the myelinated axons had severe degeneration, although the degree of the ultrastructural grade was better than in the 70 °C group [9]. Furthermore, Smith et al., in a preclinical study, stated that nerve destruction via RF current occurred at temperatures ranging from 45 to 85 °C, suggesting that effective outcomes could be achieved even at lower temperatures. [10] A previous clinical report on trigeminal neuralgia suggested that RFT at lower temperatures was associated with reduced risk of complications such as numbness and masticatory atonia, while still achieving comparable efficacy to RFT at high temperatures [11]. However, there remains no consensus regarding the optimal temperature. Therefore, future randomized controlled trials are warranted to elucidate the utility and indications of RFT.

In conclusion, we report a case of successful RFT of the mandibular nerve for pain caused by medication-related osteonecrosis of the jaw, with no symptom recurrence for 3 years. Minimally invasive RFT may have long-term effects in patients with chronic pain induced by medication-related osteonecrosis of the jaw that is refractory to conservative treatment.

## Data Availability

Not applicable.

## Abbreviations

NRS: Numerical rating scale
RFT: Radiofrequency thermocoagulation
